# Impact of Family Functioning on Adolescent Materialism and Egocentrism in Mainland China: Positive Youth Development Attributes as a Mediator

**DOI:** 10.3390/ijerph191711038

**Published:** 2022-09-03

**Authors:** Daniel Tan Lei Shek, Kim Hung Leung, Diya Dou, Xiaoqin Zhu

**Affiliations:** Department of Applied Social Sciences, The Hong Kong Polytechnic University, Hong Kong, China

**Keywords:** family functioning, positive youth development, materialism, egocentrism, Chinese adolescents, mediator

## Abstract

Although adolescent materialism and egocentrism are growing problems in Chinese societies, there are very few studies investigating their predictors and related mediators. Longitudinal studies in this area are also sparse. Based on a short-term longitudinal study (n = 4981), we assessed the impact of family functioning on materialism and egocentrism of Chinese adolescents, with positive youth development attributes as a hypothesized mediating factor. Results showed that family functioning positively predicted positive youth development attributes but negatively predicted adolescent materialism and egocentrism; positive youth development attributes also negatively predicted adolescent materialism and egocentrism. Mediational analyses showed that positive youth development attributes mediated the impact of family functioning on adolescent materialism and egocentrism. The theoretical and methodological advances of the study are discussed.

## 1. Introduction

Industrialization and modernization have brought about a growing emphasis on Western values, including individualism, materialism, and hedonism in many parts of the world. As stated by Ioane [1], consumption and materialism are regarded as universally accepted notions of well-being. Some people aspire to possess consumer goods with a high degree of aesthetic beauty and qualities that highlight health, physical beauty, satisfaction, and affluence. With particular reference to China, many Chinese consumers pursue happiness through possessing goods with luxury brands. In addition, children in China are commonly given more attention, comfort, and opportunities by their parents after the implementation of one-child policy. As a result, egocentrism and materialism of children and adolescents are prevalent in China [2] deserving further scientific investigation. 

Past research has illustrated the adverse impact of materialism and egocentrism on adolescent developmental outcomes. Materialism has been found to associate with problem and risky behaviors of adolescents such as compensated dating, compulsive buying, and problematic smartphone use (e.g., [3,4,5]). In addition, materialism leads to poor mental health and lower well-being of adolescents such as stress, depression, and less life satisfaction (e.g., [6,7]). In the same vein, the negative effects of egocentrism on adolescent risk behaviors, stress, interpersonal relationship, and conflict resolution ability have been revealed (e.g., [8,9,10]). However, in a review of the relationship between materialism and well-being, we observed that there were only nine studies based on adolescents (e.g., [11]). In the Chinese context, only two studies have been performed to assess the effects of materialism and egocentrism on adolescent delinquency. In addition, there are few studies on the antecedents of adolescent materialism and egocentrism. As such, we need to understand more about the theoretical mechanisms involved in adolescent materialism and egocentrism, particularly the related predictors.

According to Theokas and Lerner’s [12] model of ecological assets for human development, personal resources (e.g., strengths, skills, talents, and abilities) and environmental factors (e.g., family communication and support) and the dynamic interactions between them are important to adolescent development. Regarding the social context of adolescent development, the significance of the family influences on adolescent materialism and egocentrism has been emphasized in the Western studies (e.g., [13,14]). 

With specific focus on Chinese people, the impact of personal attributes, such as resilience [15] and empathy [16], and family factors such as material parenting [17] and overparenting [18] on adolescent materialism and egocentrism have been conducted. However, most studies are cross-sectional. The relationships amongst family functioning, positive youth development attributes, materialism and egocentrism of Chinese adolescents have not been fully explored. With reference to these research gaps, we examined the impact of family functioning (interpersonal resource) and positive youth development (PYD) attributes (intrapersonal resource) on materialism and egocentrism of Chinese adolescents in a 2-Wave longitudinal study using PYD attributes as a mediator.

### 1.1. Literature Review

To establish and examine the mediation model of the present investigation, each of the family functioning and PYD attributes must relate to materialism and egocentrism of adolescents. As such, we reviewed the studies on the relations between family functioning and materialism of adolescents, family functioning and adolescent egocentrism, PYD attributes and materialism of adolescents, as well as PYD attributes and adolescent egocentrism first. Afterwards, extant literature about the mediating effect of PYD attributes in the links between family functioning, and materialism and egocentrism of adolescents was further explored. 

#### 1.1.1. Family Functioning and Adolescent Materialism

The family is regarded as one of the vital socializing agents of the development of children and adolescents (e.g., [19,20]). With reference to family functioning, it reflects the quality of family environment and communications among family members [21]. A high level of family functioning is significant to healthy development of adolescents [22,23,24]. The notion is particularly relevant to China where the family is closely associated with the development of adolescents [25,26,27].

Based on Blázquez and Bonás’s [28] conceptual framework and Richins’s [29] developmental model of materialism, family environment and parenting styles are important family processes shaping adolescent materialism. Previous research has revealed the impact of the family on adolescent materialism such as the adverse effects of family conflict [13], materialism role models from parents and peers [14], in addition to high internet usage and family socio-oriented communication pattern [30] on the development of materialistic values in adolescents. 

Unfortunately, research on the family influence on materialism of Chinese adolescents is limited. Jiang et al. [31] utilized a longitudinal research design to examine the change of materialism of 738 Chinese college students and found that low family socio-economic status promoted materialism of students. Also, Li et al. [17] investigated how materialistic parenting influenced materialism of 1173 Chinese adolescents and revealed the positive association between materialistic parenting and adolescent materialism.

#### 1.1.2. Family Functioning and Adolescent Egocentrism

Similar to materialism, egocentrism is another developmental outcome of adolescents which is influenced by family functioning (e.g., [18]). Egocentrism is generally viewed as the inability to differentiate in some areas of subject–object interaction and construct relations [32]. Based on Lapsley’s [33] theory of egocentrism, adolescent egocentrism is closely related to the family environment as poorly differentiated families may over-emphasize the importance of becoming an individuated self to adolescents, which contributes to adolescents’ narcissistic isolation risk. In contrast, well-differentiated families support the appropriate balance between individuation and connectedness to the family to adolescents. To our best knowledge, research on the effect of the family on egocentrism of adolescents is very scant. Leung et al. [18] found that the conflict between the father and his child intensified the negative impact of overparenting on egocentrism of adolescents. In addition, Kauten et al. [34] indicated that parent–child conflict and marital discord increased aggressive acts of narcissistic youth or adolescents with callous–unemotional attributes. 

#### 1.1.3. Positive Youth Development and Adolescent Materialism

Under the “deficit” perspective of adolescence, pathology and problems of adolescents are the major issues for investigation. Nevertheless, the positive youth development approach focuses on positive development and healthy personal growth of adolescents [35]. Different PYD approaches such as Peter Benson’s theory and Richard Lerner’s model emphasize the significance of PYD attributes in enhancing the development of adolescents [35]. 

The relationship between positive youth development attributes and adolescent materialism is best understood in terms of the motivational theory of materialism [36]. This theory highlights the inability to fulfill psychological needs such as favorable sense of self and close interpersonal relationship as the root to the emergence of materialism. In addition, with reference to self-determination theory [37], acquired needs theory [38], and Maslow’s needs hierarchy [39], three types of needs are related to adolescent materialism (see [36]). They include tangible needs, self needs, and social needs. These needs correspond to different positive youth development attributes such as social competence (social need), cognitive competence (self need), and self-determination (self need). When one or more of these core needs are not fulfilled, adolescents may feel insecure and try to eliminate this psychological angst through material objects. For instance, adolescents who feel inadequate in their self-image may try to compensate by purchasing a high-status smartphone. As such, the PYD approach generally predicts the inverse association between PYD attributes and materialism in adolescents.

Previous research has provided empirical evidence concerning the reduction of adolescent materialism through promoting positive youth development attributes in adolescents. Geng et al. [15] revealed not only the inverse relationship between resilience and materialism, but also the buffering effect of resilience in the link between materialism and cyberbullying perpetration among adolescents. Moreover, Li et al. [40] showed the negative association between self-esteem and materialistic tendencies of adolescents, and the adverse effect of poor social class on materialism of adolescents via diminishing self-esteem. It is noteworthy that not many PYD attributes have been considered in this area.

#### 1.1.4. Positive Youth Development and Adolescent Egocentrism

Based on Elkind’s [41] cognitive model and Lapsley and Murphy’s [42] perspective-taking model of adolescent egocentrism, promotion of positive youth development attributes in adolescents, such as cognitive, social, and moral competencies, could assist adolescents in reducing egocentric bias. Past research has offered empirical evidence on the reduction of egocentrism via promotion of positive youth development attributes in adolescents and young adults. Macháčková et al. [43] showed that there was a decrease in egocentrism and other negative personal characteristics (e.g., aggression) of adolescents after enhancing their prosocial behaviors. In addition, egocentrism has been found to be ameliorated by enhancing perspective-taking ability [44] and objective self-awareness [45] of undergraduates. Nevertheless, research on the linkage between positive youth development attributes and egocentrism of Chinese adolescents is very scant.

#### 1.1.5. Positive Youth Development as a Mediator in the Linkages from Family Functioning to Adolescent Materialism and Egocentrism 

With reference to Theokas and Lerner’s [12] model of ecological assets for human development, the dynamic interplay between personal and environmental resources are crucial to the developmental outcomes of adolescents such as materialism and egocentrism. Previous research has illustrated that positive youth development attributes (personal resources) and family functioning (environmental resource) are closely related to each other. Specifically, family functioning contributes to the development of positive youth development attributes. For example, Yu et al. [24] showed that family functioning positively predicted developmental assets characterized by resilience, self-efficacy, optimism, and hope of Chinese students. Moreover, family cohesion and adaptability positively contributed to psychological capital, which in turn negatively predicted academic burnout of students. Shek et al. [46] found that the quality of parent–child relationship predicted meaning of life in Chinese adolescents in a positive manner. Shek et al. [47] also showed the positive association between family functioning and a wide range of positive youth development attributes.

To our best knowledge, research about the influence of family functioning on adolescent materialism and egocentrism with positive youth development attributes as a mediator is lacking. Trzcińska and Sekścińska [48] only assessed the impact of household financial status on materialism in 1138 Polish adult workers. The findings revealed that monthly income of the family (objective financial status) positively contributed to self-esteem, which in turn negatively predicted the centrality dimension of materialism of the workers. 

### 1.2. The Present Study

Extant literature concerning the impact of family functioning and PYD attributes on materialism and egocentrism in Chinese adolescents is subject to several limitations. First, research targeted at investigating family functioning and PYD attributes as predictors of adolescent materialism and egocentrism is sparse. Second, no study has been conducted to examine the mediating role of PYD attributes in the family functioning-adolescent materialism and family functioning-adolescent egocentrism links. Third, longitudinal studies in this field are very scant. Fourth, the number of participants in some studies is not large (e.g., [15,31]), thus restricting the generalizability of research findings. Fifth, psychometrically sound measures to a wide range of PYD attributes are sparse even though there are discrete scales to assess individual PYD attributes (e.g., resilience). With reference to these weaknesses, we examined the relationships between family functioning and PYD attributes, and materialism and egocentrism of adolescents, in addition to the mediating role of PYD attributes in the links from family functioning to materialism and egocentrism of adolescents. Six hypotheses of this study are proposed as follows:

**Hypothesis** **1.** *Family functioning would be inversely associated with adolescent materialism (see [13,17]).*

**Hypothesis** **2.** *Family functioning would be inversely correlated with egocentrism of adolescents (see [18,33]).*

**Hypothesis** **3.** *PYD attributes would be inversely correlated with adolescent materialism (see [15,35,40]).*

**Hypothesis** **4.** *PYD attributes would be inversely correlated with adolescent egocentrism (see [35,43,44]).*

**Hypothesis** **5.** *PYD attributes would mediate the link from family functioning to materialism of adolescents (see [48]).*

**Hypothesis** **6.** *PYD attributes would mediate the link from family functioning to egocentrism of adolescents (see [18,43,46]).*

The present investigation is a part of a larger project investigating adolescent development and well-being among Chinese adolescents in Hong Kong and mainland China. Specifically, the effects of family processes such as family functioning (contextual factor) and positive youth development attributes (intrapersonal factor) on youth development and well-being of adolescents were examined. The dataset used in the present study was the same as those used in a recently published paper [47] but with different research foci. In a previously published paper [47], we assessed the impact of family functioning and positive youth development attributes on delinquency of adolescents (i.e., antisocial behaviors) using positive youth development as the mediator. The paper was included in a special issue on psycho-criminology. In this paper, we investigated a different theoretical model to explore the effects of family functioning and positive youth development attributes on adolescent materialism (belief about materialism) and egocentrism (social-psychological construct). Theoretically, PYD has a significant mediating effect on the links from family functioning to different developmental outcomes of adolescents. As such, we asked different research questions with reference to different outcome variables in this study. Using the same dataset to address different research questions and published different papers is a common practice in longitudinal studies. For examples, same dataset was used in the following pairs of articles: [49] and [50]; [51] and [52]; [53] and [54]).

## 2. Materials and Methods

### 2.1. Participants and Procedures

In the present investigation, 5690 adolescents from five randomly selected high schools in Sichuan, China participated in the study in 2019 before the outbreak of COVID-19 (Wave 1). After six months, these adolescents took part in the study again after school resumption (Wave 2) (*n* = 4981). As we could not match the responses at Wave 1 and Wave 2 of some participants (only names, classes, and class numbers of students as identifiers for matching), the final sample was composed of 4922 participants who studied from grade three to grade nine. There were 2387 males (48.5%) and 2535 females (51.5%), with an average age of 13.2 years old (SD = 1.32) at Wave 1. Most of the students were Hans people (99.3%) who are native Chinese and speak different varieties of the Chinese language. Nearly 60% of students resided in cities. The attrition rate was 12.5%.

Before collecting the data, we sought for research ethics approval from Sichuan University and informed consent from schools, parents, and students. Respondents answered the same questionnaire in class at Wave 1 and Wave 2 (see [55] for more details of the demography of the participants and the study procedures). Aligned with the research questions in adolescents, the responses of adolescents aged 11 and above were the major concern of this study.

### 2.2. Instruments

In the survey, we adopted different scales to assess psychosocial adaptation of adolescents. Aligned with the objective of this study, we only addressed the relationships amongst family functioning, positive youth development, materialism, and egocentrism. Participants completed the same questionnaire at two waves six months apart.

#### 2.2.1. Family Functioning

A 33-item Chinese Family Assessment Instrument (C-FAI) was utilized to examine five different aspects of family functioning [56] such as mutuality, communication, and parental control. For each item, participants answered on a 5-point scale ranging from 1 (most similar) to 5 (most dissimilar). Scores were reversed so that a greater score implies better family’s functioning. C-FAI has been used in previous research using Chinese adolescents as samples and has been found to possess good psychometric properties (e.g., [57,58]).

#### 2.2.2. Positive Youth Development

In this study, positive attributes of adolescents were assessed via the 80-item Chinese Positive Youth Development Scale (CPYDS) [59,60]. The positive attributes are categorized into fifteen dimensions including healthy relationship with adults, psychosocial competencies, moral competence, self-determination, etc. All items were rated on a 6-point scale ranging from 1 (strongly disagree) to 6 (strongly agree), except for spirituality (1 = most negative to 7 = most positive). A greater score implies a greater degree of positive youth development in this study. Past studies with Chinese adolescents have illustrated that CPYDS possesses good psychometric properties (e.g., [61,62]). 

#### 2.2.3. Materialism

The Chinese Adolescent Materialism Scale (CAMS) was adopted to examine the materialism of Chinese adolescents in this study [63]. It is a 19-item scale reflecting three dimensions including acquisition centrality, hedonistic pursuit, and materialistic possession. Participants responded to each item on a 6-point scale ranging from 1 (strongly disagree) to 6 (strongly agree). A greater score means a greater degree of materialism in this study. Past research using Chinese samples has indicated that CAMS has good validity and reliability (e.g., [63,64]). 

#### 2.2.4. Egocentrism

The Chinese Adolescent Egocentrism Scale (CAES) was utilized to examine egocentrism of Chinese adolescents in the present investigation [16]. It is a 14-item scale reflecting two dimensions including “self-conceit” and “self over others”. Participants rated each item on a 6-point scale ranging from 1 (strongly disagree) to 6 (strongly agree). A greater score indicates a greater level of egocentrism in this study. Past studies with Chinese adolescents have shown that CAES has good psychometric properties [16,64].

### 2.3. Data Analysis

**Figure 1:**
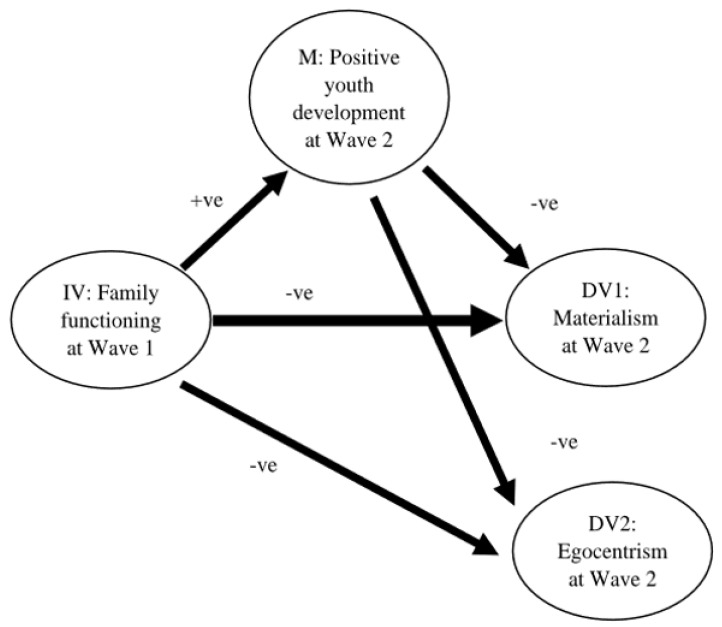
Conceptual model for the mediating effect of positive youth development at Wave 2 on the paths from family functioning at Wave 1 to materialism and egocentrism at Wave 2. Note. IV = independent variable, M = mediator, DV = dependent variable, +ve = hypothesized positive relationship, −ve = hypothesized negative relationship.

In this study, the relationships among the second-order latent factors of family functioning, positive youth development, materialism, and egocentrism were assessed using structural equation modeling (SEM). Based on Baron and Kenny’s [65] analytic procedures for mediation, three essential conditions for the development of the mediation paths of materialism and egocentrism were examined individually first. The conditions for materialism include the effects from Wave 1 family functioning (predictor variable) on materialism at Wave 2 (dependent variable), from Wave 1 family functioning to Wave 2 positive youth development (mediator), and from Wave 2 positive youth development to Wave 2 materialism when regressing Wave 2 materialism on both Wave 1 family functioning and Wave 2 positive youth development. The same conditions using egocentrism as the dependent variable were also tested. As all effects were significant, it was justified to create the mediation paths from Wave 1 family functioning to Wave 2 materialism and egocentrism via Wave 2 positive youth development in the overall SEM model (Figure 1). This overall model was tested to reveal the mediating effects of positive youth development on the linkages from family functioning to materialism and egocentrism of Chinese adolescents.

Based on [66], we assessed the measurement part of the SEM model first before exploring its structural part in this study. The measurement model was specified with the constructs being inferred by their corresponding observed indicators without correlated uniqueness. In addition, correlations among all latent constructs were estimated. After confirming the validity of the measurement portion, the inter-relationships among latent variables were examined. Hypothesized predictions among the latent variables were specified in the structural portion of the SEM model.

We utilized maximum likelihood estimation to examine the SEM model via LISREL 8.54. Adopting the criteria stated in Brown [67], standardized root-mean-square residual (SRMR) less than 0.08 indicates a good fit of the model [68]. Moreover, root-mean-square error of approximation (RMSEA) less than 0.05, between 0.05 and 0.08, and between 0.08 and 0.10 indicate a close fit, a fair fit, and a mediocre fit, respectively [69]. In addition, non-normed fit index (NNFI) and comparative fit index (CFI) more than 0.90 indicate an acceptable fit [70]. As the sample size of this study was large, which was likely to render lower standards errors to the estimates [71], we used the Sobel test to assess the level of significance of indirect effects. The indirect effect is regarded as significant if no zero is included between the lower and upper bounds of 95% CI for indirect effect estimates [72].

In this study, we treated age and gender as covariates and they were controlled for in the analyses. In particular, the effects of covariates on positive youth development, materialism, and egocentrism at Wave 2 were assessed (see [73]). Age and gender were regarded as covariates because they have been revealed to associate with positive youth development, materialism, and egocentrism in previous related studies (e.g., [64,74]). In the present study, significant effects of age and gender on Wave 2 materialism (age: β = 0.22, *p* < 0.001; gender: β = 0.13, *p* < 0.001), Wave 2 egocentrism (age: β = 0.07, *p* < 0.001; gender: β = 0.06, *p* < 0.05), and Wave 2 positive youth development (age: β = −0.09, *p* < 0.001; gender: β = 0.08, *p* < 0.001) were also revealed using multiple regression analyses. Specifically, female adolescents had higher levels of materialism and egocentrism than male counterparts because of lower self-concept and lower self-esteem in female adolescents (see [64]). As such, the impact of age and gender on the mediating effects of positive youth development in the paths from family functioning to materialism and egocentrism links was controlled for.

## 3. Results

### 3.1. Attrition Analyses

In this study, the problem of participant dropout was not a major issue because sample attrition was only 12.5%. Moreover, there was no difference in demographic features like gender and age between the matched sample and the dropouts. The Little’s MCAR test result (χ2(290) = 104.21, *p* = 1.000) also indicated that the missing values of the dataset were completely at random [75].

### 3.2. Descriptive Statistics, Reliability, and Inter-Correlation

Table 1 shows descriptive statistics and reliability of all variables, and correlations among them. As Cronbach’s alpha coefficients ranged from 0.67 to 0.98, all scales were reliable (see [76,77]). As expected, Wave 1 family functioning and Wave 2 positive youth development were inversely correlated with materialism and egocentrism at Wave 2. Moreover, Wave 1 family functioning was positively correlated with Wave 2 positive youth development. In addition, Wave 2 materialism was positively related to Wave 2 egocentrism. We also found that there were gender differences in materialism and egocentrism at Wave 1 and Wave 2 (*p* < 0.05) with females showing higher levels on both measures.

### 3.3. Scale Validation

The first- and second-order factorial structures of CAMS and CAES were tested by CFA in the present study.

#### 3.3.1. Chinese Family Assessment Instrument and Chinese Positive Youth Development Scale

In a previous study [47], we validated the five-factor and fifteen-factor structures of C-FAI and CPYDS, respectively. These two scales were valid and reliable. Nevertheless, it is noteworthy that the RMSEA and SRMR values in the C-FAI model represented fair fit only (but they are still acceptable), although NNFI and CFI indices showed excellent fit.

#### 3.3.2. Chinese Adolescent Materialism Scale

The three-factor structure of CAMS with two covariances between errors was supported (χ2 = 6885.93, df = 147, *p* < 0.001; NNFI = 0.96, CFI = 0.97, RMSEA = 0.105, SRMR = 0.062) in the present study. Factor loadings were statistically significant at a 0.05 level with the values ranging from 0.59 to 0.83. The subscales exhibited high reliability because all composite reliabilities (CRs) were above 0.70, nearly all average variance extracted (AVEs) were above 0.50, and mean inter-factor correlations were high (r = 0.88). Moreover, the higher-order CFA results further supported the second-order structure of CAMS (χ2 = 6885.93, df = 147, *p* < 0.001; NNFI = 0.96, CFI = 0.97, RMSEA = 0.105, SRMR = 0.062). All loadings of the second-order factor (materialism) on three primary factors (acquisition centrality, materialistic possession, and hedonistic pursuit) were above 0.80 (*p* < 0.05). The reliability of the total scale of materialism was 0.95. Although NNFI and CFI indices showed excellent fit, RMSEA values in these two models provided fair fit only.

#### 3.3.3. Chinese Adolescent Egocentrism Scale

The factorial validity of two-factor structure of CAES with one error covariance was supported in this study (χ2 = 3233.13, df = 75, *p* < 0.001; NNFI = 0.94, CFI = 0.95, RMSEA = 0.093, SRMR = 0.090). Factor loadings ranged from 0.59 to 0.83 (*p* < 0.05). The reliability of the subscales was good because mean CR was 0.83, nearly all AVEs were above 0.50, and the average inter-factor correlation was moderate (r = 0.40). In addition, the second-order structure of CAES (χ2 = 3233.13, df = 75, *p* < 0.001; NNFI = 0.94, CFI = 0.95, RMSEA = 0.093, SRMR = 0.090) was confirmed. All loadings of the second-order factor (egocentrism) on two primary factors (self-conceit and self over others) were above 0.50 (*p* < 0.05). The reliability of the total scale of egocentrism was 0.85. Nevertheless, while NNFI and CFI values were very good in these two models, RMSEA and SRMR values suggested fair fit only.

### 3.4. Prediction and Mediation Analyses

#### 3.4.1. Predictive Effects of Family Functioning and Positive Youth Development on Materialism

Following Baron and Kenny’s [65] analytic approach to mediation, individual direct effects of Wave 1 family functioning on Wave 2 materialism and Wave 2 positive youth development, and the effect of Wave 2 positive youth development on Wave 2 materialism when regressing Wave 2 materialism on both Wave 1 family functioning and Wave 2 positive youth development were examined. The positive predictive effect of family functioning on positive youth development was reported in a previous study [47]. The present results revealed that Wave 1 family functioning inversely predicted Wave 2 materialism (β = −0.26, *p* < 0.001; fit indices: χ2 = 31,878.2, df = 1262, *p* < 0.001, NNFI = 0.95, CFI = 0.95, RMSEA = 0.088, SRMR = 0.076). Wave 2 positive youth development inversely contributed to Wave 2 materialism when regressing Wave 2 materialism on both Wave 1 family functioning and Wave 2 positive youth development (β = −0.31, *p* < 0.001; fit indices: χ2 = 74,955.1, df = 8483, *p* < 0.001, NNFI = 0.98, CFI = 0.98, RMSEA = 0.048, SRMR = 0.051). As such, three necessary conditions for the establishment of mediating paths for materialism were fulfilled.

#### 3.4.2. Predictive Effects of Family Functioning and Positive Youth Development on Egocentrism

The positive predictive effect of family functioning on positive youth development was reported in a previous study [47]. The present results indicated that Wave 1 family functioning negatively predicted Wave 2 egocentrism (β = −0.18, *p* < 0.001; fit indices: χ2 = 28,106.0, df = 1023, *p* < 0.001, NNFI = 0.94, CFI = 0.94, RMSEA = 0.092, SRMR = 0.093). Wave 2 positive youth development inversely contributed to Wave 2 egocentrism when regressing Wave 2 egocentrism on both Wave 1 family functioning and Wave 2 positive youth development (β = −0.08, *p* < 0.001; fit indices: χ2 = 71,772.6, df = 7846, *p* < 0.001, NNFI = 0.98, CFI = 0.98, RMSEA = 0.049, SRMR = 0.067). As such, three necessary conditions for the establishment of mediating paths for egocentrism were fulfilled.

#### 3.4.3. Mediating Effects of Positive Youth Development on the Paths from Family Functioning to Materialism and Egocentrism

In the present investigation, the CFA results of the overall SEM model illustrated that the measurement portion of the model fit the data well (χ2 = 84,172.2, df = 10,403, *p* < 0.001, NNFI = 0.98, CFI = 0.98, RMSEA = 0.046, SRMR = 0.062). This indicated that the variances of the indicators were best explained by their corresponding first-order latent variables. In addition, the variances of first-order latent variables were best explained by their corresponding second-order constructs (family functioning, positive youth development, materialism and egocentrism). Wave 1 family functioning was positively correlated with Wave 2 positive youth development (r = 0.36, *p* < 0.05), but negatively associated with Wave 2 materialism (r = −0.26, *p* < 0.05) and Wave 2 egocentrism (r = −0.22, *p* < 0.05). Wave 2 positive youth development was inversely related to Wave 2 materialism (r = −0.36, *p* < 0.05) and Wave 2 egocentrism (r = −0.18, *p* < 0.05). Wave 2 materialism was positively related to Wave 2 egocentrism (r = 0.65, *p* < 0.05).

**Figure 2:**
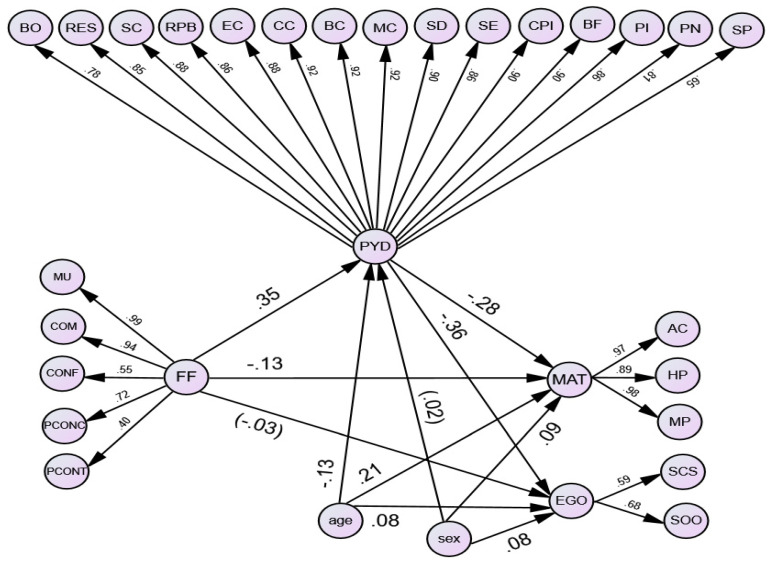
Overall SEM model of Wave 2 positive youth development on the links from Wave 1 family functioning to Wave 2 materialism and Wave 2 egocentrism controlling for age and sex. Note: All estimates are standardized ones. All paths were significant at 0.05 levels except for the path with the estimate in parenthesis. Indicators of all latent variables and correlations among the factors of family functioning are omitted for clarity. MU = mutuality, COM = communication, CONF = conflict and harmony, PCONC = parental concern, PCONT = parental control, FF = family functioning, BO = bonding, RES = resilience, SC = social competence, RPB = recognition for positive behavior, EC = emotional competence, CC = cognitive competence, BC = behavioral competence, MC = moral competence, SD = self-determination, SE = self-efficacy, CPI = clear and positive identity, BF = beliefs in the future, PI = prosocial involvement, PN = prosocial norms, SP = spirituality, PYD = positive youth development, AC = acquisition centrality, HP = hedonistic pursuit, MP = materialistic possession, MAT = materialism, SCS = self-conceit, SOO = self over others, EGO = egocentrism, Age = age, Sex = sex.

Figure 2 illustrates how family functioning, positive youth development, materialism, and egocentrism were related to each other. The present CFA results indicated a good fit of the overall SEM model to the data (χ2 = 87,809.3, df = 10,691, *p* < 0.001, NNFI = 0.98, CFI = 0.98, RMSEA = 0.046, SRMR = 0.069). Wave 1 family functioning positively predicted Wave 2 positive youth development (β = 0.35, *p* < 0.05), but negatively predicted Wave 2 materialism (β = −0.13, *p* < 0.05). However, it did not significantly predict Wave 2 egocentrism (β = −0.03, *p* = 0.09). Wave 2 positive youth development negatively predicted Wave 2 materialism (β = −0.28, *p* < 0.05) and Wave 2 egocentrism (β = −0.36, *p* < 0.05).

Three types of effects for the mediation paths to materialism and egocentrism in the holistic SEM model are shown in Table 2. The direct effect of Wave 1 family functioning on Wave 2 materialism reduced after incorporating Wave 2 positive youth development (β = −0.128, *p* < 0.05) into the analysis, compared to the direct effect without including positive youth development (β = −0.226, *p* < 0.05). This illustrates the partial mediating role of Wave 2 positive youth development in the path from Wave 1 family functioning to Wave 2 materialism (see [65]). The indirect effect of Wave 1 family functioning on Wave 2 materialism through Wave 2 positive youth development was negative (β = −0.098, *p* < 0.05; 95% CI = −0.118 to −0.078). It explained 43.4% of the total effect. In sum, the partial mediation model explained 19.8% of the variance in Wave 2 materialism, which was greater than those explained by the direct effect model without positive youth development (6.5%). As such, Hypotheses 1, 3, and 5 were supported.

Regarding egocentrism, the present results showed a negative indirect effect of Wave 1 family functioning on Wave 2 egocentrism via Wave 2 positive youth development (β = −0.124, *p* < 0.05; 95% CI = −0.177 to −0.071). Notably, when Wave 2 positive youth development was included in the model, the contribution from Wave 1 family functioning to Wave 2 egocentrism became nonsignificant (β = −0.028, *p* = 0.09), which revealed that positive youth development fully mediated the impact of family functioning on egocentrism (see [65]). In sum, the full mediation model explained 15.7% of the variance in Wave 2 egocentrism, which was more than those explained by the direct effect model without positive youth development (3.2%). The findings supported Hypotheses 4 and 6.

## 4. Discussion

In the light of the lack of systematic research on the relationships amongst family functioning, positive youth development attributes, adolescent materialism and egocentrism, the present study responded to this research gap and took the mediating role of positive youth development attributes in the linkages between family functioning, and adolescent materialism and egocentrism into account. Although some theories highlight the role of personal and social resources in adolescent materialism and egocentrism (e.g., [28,33]), studies on the antecedents of materialism and egocentrism mainly covered either type of resources. Consequently, this study makes the theoretical advance in this area through the development of the holistic model to understand the joint impact of family functioning and positive youth development attributes on materialism and egocentrism of adolescents.

Moreover, this study makes several methodological advances. First, since past research on the linkages amongst family functioning, positive youth development, and adolescent materialism and egocentrism involved only one or a few aspects of family functioning and positive youth development (e.g., [13,15,18,78]), we used more dimensions of family functioning and positive youth development in this study. Second, as longitudinal research in this area is limited in China, we utilized a 2-wave longitudinal design accompanied with large Chinese samples in this study.

With reference to the question about the relationship between family functioning and materialism of adolescents, the present findings illustrated that family functioning inversely predicted materialism of adolescents over time. As such, Hypothesis 1 was verified. The role of family in the prevention of adolescents from developing materialistic beliefs is highlighted (e.g., [17]). In addition, it is in line with existing family functioning models (e.g., [14,23]). As stated by Richins [29], supportive parents offer greater personal security to their children and these children will subsequently place a lower emphasis on material possession. Moreover, a supportive parent–child relationship would help children resist peers’ demands for material conformity. In contrast, parental rejection would result in the sense of insecurity and inferiority that put the child at risk for materialism. Since previous studies have mainly operationalized family functioning as a unidimensional construct, such as communication patterns within the family (e.g., [30]) or parenting style (e.g., [17]), we operationalized family functioning as the composite of different aspects of family interaction and parenting styles, which is consistent with the notion of the multi-dimensionality of family functioning [79].

In response to the question about the relationship between family functioning and egocentrism of adolescents, the findings of the present study did not support Hypothesis 2 that family functioning inversely contributed to egocentrism of adolescents over time. We found that family functioning did not significantly relate to adolescent egocentrism after taking the influence of positive youth development attributes into account. It might be best explained by full mediation exerted by positive youth development attributes on the family functioning-adolescent egocentrism link. That is, the effect from family functioning on adolescent egocentrism is 100% mediated by positive youth development attributes. The family functioning-adolescent egocentrism pathway is totally broken so that family functioning has no direct effect on adolescent egocentrism [80]. In fact, without incorporating PYD attributes or other intervening factors as the mediators in the relationship between family functioning and adolescent egocentrism, our finding on the influence of family functioning on adolescent egocentrism was consistent with previous studies (e.g., [18]) that family functioning contributed negatively to adolescent egocentrism. As such, the protective role of the family in eliminating adolescents’ egocentric bias is revealed. Regarding the positive contribution of family functioning to positive youth development attributes over time, Shek et al. [47] has already revealed that family functioning contributed to positive youth development attributes in a favorable manner. As we used same dataset in this paper, it is not surprising to see that there was a positive relationship between family functioning and positive youth development attributes.

Regarding the question about the relation between PYD attributes and materialism of adolescents, our findings revealed negative prediction of PYD attributes to adolescent materialism. It supported Hypothesis 3. This finding is consistent with Burroughs’s [36] motivational theory of materialism that adolescents who do not fulfill their social and self needs may place greater values on material possession to ameliorate their sense of insecurity and inferiority. It also offers empirical evidence to the basic notion of the positive youth development approach which emphasizes the importance of developmental assets in protecting adolescents from negative developmental outcomes [35].

With reference to the question of the relation between PYD attributes and egocentrism of adolescents, our findings indicated a negative contribution of PYD attributes to adolescent egocentrism and hence verified Hypothesis 4. This finding is consistent with Elkind’s [41] cognitive model and Lapsley and Murphy’s [42] perspective-taking model of adolescent egocentrism which advocate that positive youth development attributes would reduce egocentrism of adolescents. It also echoes the findings by Golubickis et al.’s [44] study which provide empirical support to the reduction of egocentrism via the promotion of third-person perspective of self to students. As previous studies concerning the influence of PYD attributes on adolescent egocentrism have focused on one or few positive attributes such as resilience (e.g., [15]) and self-esteem (e.g., [40]), the present investigation advanced in the knowledge of this area by examining the aggregation of PYD attributes from different domains such as moral competence, self-efficacy and spirituality of adolescents [59]. Even though we did not analyze the effect of each individual PYD attribute, the results of higher-order CFA illustrated that the second-order latent construct of PYD attributes was best inferred by all of the 15 dimensions of PYD. As such, the whole-person development of adolescents is considered in this study. To understand the significance of each PYD attribute in the relations between family functioning, and materialism and egocentrism of adolescents, future research might investigate the impact of each PYD attribute in the relations between family functioning, and adolescent materialism and egocentrism separately.

Finally, regarding the question of the importance of PYD attributes in the relationships between family functioning, and materialism and egocentrism of adolescents, our findings revealed the mediating role of PYD attributes inthe linkages between family functioning, and adolescent materialism as well as egocentrism (Hypothesis 5 and Hypothesis 6). First, the impact of family functioning on adolescent materialism over time was partially mediated by positive youth development attributes. This observation partially supported Hypothesis 5. It echoes Theokas and Lerner’s [12] model of ecological assets for human development that developmental outcomes of adolescents like materialism and egocentrism are influenced by dynamic interplay between personal and social resources available to adolescents. On the other hand, the effect of family functioning on egocentrism of adolescents over time was fully mediated by positive youth development attributes. This finding supported Hypothesis 6 and further highlighted the importance of dynamic interaction of personal and social resources on adolescent development stated in Theokas and Lerner’s [12] model of ecological assets for human development. That is, healthy families with good communication amongst family members and positive reciprocity would foster positive youth development attributes of adolescents, which in turn result in preventing adolescents from believing that other people are preoccupied with their appearance and behaviors. To our best knowledge, the mediating role of positive youth development attributes in the family functioning-adolescent materialism and family functioning-adolescent egocentrism links has not been explored. As such, our findings are pioneering and valuable. In addition, in line with the findings reported by [47], the present investigation suggests that the extent of the mediating effect of positive youth development depends on the nature of the outcome variables.

## 5. Implications

### 5.1. Theoretical Implications

There are several implications of this study. Conceptually, the protective role of family functioning in materialism and egocentrism of adolescents in mainland China is emphasized. Although different theories (e.g., [28,29,33]) emphasize the significance of the influence of family on the development of materialism, the findings in this area are mixed (e.g., [20,81,82]). Our findings based on longitudinal data from the large sample provide empirical evidence to the inverse relationship between family functioning and adolescent materialism, which in turn support family theories of materialism (e.g., [28,29]). This echoes Shrum et al.’s [83] review article that greater focus of longitudinal and causal methods is one of the ways for making important advances for materialism research, apart from larger emphasis on self-concept clarity and better understanding on how materialism is harmful or beneficial to adolescents. Moreover, based on Theokas and Lerner’s [12] model of ecological assets for human development, both personal and social resources are crucial to the development of adolescent materialism and egocentrism. Nonetheless, no research has systematically examined the impact of dynamic interplay between family functioning (social factor) and positive youth development attributes (personal factor) on materialism and egocentrism. As such, the present study advances in this area by developing theoretical models to understand how positive youth development attributes contribute to the mechanism in the linkages from family functioning to adolescent materialism and egocentrism. In addition, the findings of our previous paper have revealed significant influence of family functioning and positive youth development attributes on adolescent delinquency [47]. As materialism and egocentrism have been found to contribute significantly to adolescent delinquency (e.g., [47,84]), the findings of this paper may help us develop a more thorough understanding about the beneficial influence of good family functioning on the reduction of delinquency of adolescents. That is, family functioning and positive youth attributes may assist in preventing delinquency of adolescents via reducing materialism and egocentrism of adolescents. As we only collected the data at two different points of time in this study, we need data across multiple points of time to examine this possibility. In short, the present study broadens our understanding of the influence of family functioning and positive youth development attributes on the behavior in the social-psychological domains of adolescents in terms of materialistic beliefs and perceived egocentric attributes. However, the results of this study could be interpreted with caution because the measures to adolescent materialism and egocentrism only fit fairly to the data.

### 5.2. Practical Implications

Practically, the findings of the present investigation advocate that materialism and egocentrism of adolescents could be eliminated by specialized intervention programs in family functioning and positive youth development. In the information technology era, mass media have played a pivotal role in influencing materialism and egocentrism of adolescents (e.g., [81,85]). However, under the Confucian tradition in China, parents still exert great influence on the developmental outcomes of adolescents who strongly attach the value of filial piety (see [1]). As indicated by Behal and Soni’s [30], young online media users are often more materialistic because parents do not control the internet usage of their children and correct their children’ desire of buying excessive goods, and hence facilitate the development of their materialistic values and beliefs. As such, intervention targeted at assisting parents in imposing proper control of media habits of their children (by imposing time and content limitation) and developing concept-oriented communication would reduce adolescents’ materialistic values and egocentric bias [86].

In addition, the findings suggest that promotion of positive development attributes to adolescents would reduce adolescents from developing egocentric bias (e.g., [43,44]). In their discussion of the significant findings on the influence of positive youth development attributes on delinquency of adolescents, Shek et al. [47] already made this recommendation. Nevertheless, research-validated holistic positive youth development programs to adolescents in China are limited [87]. As such, we developed the Tin Ka Ping P.A.T.H.S. Program in China based on the favorable impact of the Project P.A.T.H.S. in Hong Kong. Zhu and Shek [88] carried out pioneer research to evaluate the impact of a positive youth development program (“Tin Ka Ping P.A.T.H.S. Project”) on satisfaction with life, delinquent acts, and depression of 1044 adolescents before and after participating in the program in mainland China. They revealed a significant promotion in PYD attributes and reduction in delinquent acts of the experimental group, but not in control counterparts. As such, this project could be used as an option to promote PYD attributes and reduce adolescent materialism and egocentrism in mainland China.

## 6. Limitations

There are several shortcomings of this study. First, since the data were only gathered at two different points of time, the results of the causal impact of the mediator should be viewed as preliminary. Researchers should gather more longitudinal data to fully understand the mediating effect of PYD attributes. Second, as the participants of this study were restricted to adolescents in Sichuan, China, we do not know whether the findings could be generalized to adolescents in other Chinese countries. Future research should conduct the same investigation in other Chinese communities to increase the validity of the findings of the present study. Finally, as we only gathered self-reported data in this study, bias due to a common method variance may exist. Future research should collect data from different sources such as parents’ rating on materialism and egocentrism of their children.

## 7. Conclusions

In response to the shortcomings of past studies on the prevention of materialism and egocentrism of adolescents in mainland China, there are several theoretical and methodological advances in this study. Theoretically, instead of using the deficit’s perspective, we adopted a positive approach and assessed the protective functions of family functioning and PYD attributes in materialism and egocentrism of adolescents. Methodologically, we carried out a longitudinal study utilizing greater sample size and psychometrically sound instruments. As expected, this study illustrates that family functioning prevents adolescents from developing materialism and egocentrism by the promotion of adolescents’ positive attributes. Consequently, any intervention measure to adolescent materialism and egocentrism would take both family contexts and adolescents’ positive youth attributes into account.

## Figures and Tables

**Table 1:** Descriptive statistics, reliability, and inter-correlation of variables (*n* = 4922).

	1	2	3	4	5	6	7	8	9	10	11	12	13	14	15	16	17	18	19	20	21	22	23	24	25	26	27	28	29	30	31
1. Age	-																														
2. Sex ^a^	0.00	-																													
3. W1 MU	**−0.13**	0.00	-																												
4. W1 COM	**−0.15**	−0.03	**0.83**	-																											
5. W1 CONF	**−0.10**	**0.04**	**0.50**	**0.44**	-																										
6. W1 PCOC	**−0.06**	**0.08**	**0.65**	**0.58**	**0.52**	-																									
7. W1 PCOT	**−0.05**	**0.08**	**0.29**	**0.31**	**0.54**	**0.39**	-																								
8. W1 FF	**−0.12**	**0.05**	**0.83**	**0.80**	**0.77**	**0.80**	**0.69**	-																							
9. W2 BO	**−0.11**	0.00	**0.29**	**0.32**	**0.24**	**0.22**	**0.20**	**0.33**	-																						
10. W2 RES	**−0.14**	−0.02	**0.28**	**0.30**	**0.26**	**0.21**	**0.18**	**0.31**	**0.76**	-																					
11. W2 SC	**−0.14**	−0.01	**0.26**	**0.28**	**0.23**	**0.18**	**0.17**	**0.29**	**0.63**	**0.69**	-																				
12. W2 RPB	**−0.11**	0.01	**0.25**	**0.27**	**0.23**	**0.19**	**0.20**	**0.30**	**0.69**	**0.66**	**0.71**	-																			
13. W2 EC	**−0.09**	*−0.03*	**0.27**	**0.31**	**0.23**	**0.18**	**0.19**	**0.30**	**0.61**	**0.68**	**0.74**	**0.70**	-																		
14. W2 CC	**−0.18**	*−0.03*	**0.29**	**0.31**	**0.25**	**0.20**	**0.19**	**0.32**	**0.63**	**0.74**	**0.76**	**0.72**	**0.78**	-																	
15. W2 BC	**−0.14**	−0.01	**0.26**	**0.28**	**0.22**	**0.19**	**0.17**	**0.29**	**0.61**	**0.68**	**0.71**	**0.68**	**0.70**	**0.78**	-																
16. W2 MC	**−0.13**	0.03	**0.25**	**0.26**	**0.23**	**0.17**	**0.16**	**0.27**	**0.59**	**0.64**	**0.69**	**0.64**	**0.68**	**0.73**	**0.75**	-															
17. W2 SD	**−0.16**	−0.01	**0.25**	**0.28**	**0.24**	**0.19**	**0.17**	**0.29**	**0.59**	**0.69**	**0.68**	**0.62**	**0.68**	**0.76**	**0.77**	**0.74**	-														
18. W2 SE	**−0.13**	−0.02	**0.22**	**0.25**	**0.19**	**0.16**	**0.16**	**0.26**	**0.51**	**0.58**	**0.57**	**0.54**	**0.56**	**0.64**	**0.63**	**0.59**	**0.66**	-													
19. W2 CPI	**−0.18**	**−0.07**	**0.28**	**0.33**	**0.23**	**0.19**	**0.18**	**0.31**	**0.60**	**0.66**	**0.68**	**0.63**	**0.69**	**0.73**	**0.67**	**0.67**	**0.71**	**0.63**	-												
20. W2 BF	**−0.20**	−0.02	**0.28**	**0.31**	**0.25**	**0.20**	**0.19**	**0.32**	**0.55**	**0.66**	**0.64**	**0.58**	**0.63**	**0.71**	**0.65**	**0.64**	**0.70**	**0.59**	**0.80**	-											
21. W2 PI	**−0.19**	*0.03*	**0.29**	**0.30**	**0.24**	**0.19**	**0.20**	**0.31**	**0.61**	**0.63**	**0.64**	**0.67**	**0.63**	**0.70**	**0.65**	**0.67**	**0.64**	**0.57**	**0.70**	**0.70**	-										
22. W2 PN	**−0.11**	**0.08**	**0.23**	**0.23**	**0.21**	**0.19**	**0.17**	**0.26**	**0.54**	**0.58**	**0.60**	**0.61**	**0.55**	**0.64**	**0.63**	**0.63**	**0.59**	**0.51**	**0.59**	**0.61**	**0.73**	-									
23. W2 SP	**−0.13**	**−0.10**	**0.32**	**0.36**	**0.27**	**0.23**	**0.25**	**0.37**	**0.51**	**0.56**	**0.49**	**0.49**	**0.59**	**0.54**	**0.46**	**0.45**	**0.47**	**0.44**	**0.62**	**0.58**	**0.52**	**0.38**	-								
24. W2 PYD	**−0.18**	−0.02	**0.33**	**0.37**	**0.29**	**0.24**	**0.23**	**0.38**	**0.77**	**0.83**	**0.83**	**0.81**	**0.84**	**0.89**	**0.84**	**0.82**	**0.84**	**0.74**	**0.86**	**0.83**	**0.83**	**0.75**	**0.69**	-							
25. W2 AC	**0.27**	**−0.10**	**−0.20**	**−0.22**	**−0.18**	**−0.14**	**−0.15**	**−0.23**	**−0.25**	**−0.29**	**−0.25**	**−0.25**	**−0.23**	**−0.27**	**−0.24**	**−0.26**	**−0.22**	**−0.21**	**−0.27**	**−0.29**	**−0.31**	**−0.24**	**−0.31**	**−0.32**	-						
26. W2 MP	**0.27**	−0.02	**−0.22**	**−0.24**	**−0.18**	**−0.13**	**−0.16**	**−0.24**	**−0.26**	**−0.28**	**−0.26**	**−0.25**	**−0.26**	**−0.28**	**−0.24**	**−0.27**	**−0.22**	**−0.23**	**−0.30**	**−0.30**	**−0.32**	**−0.23**	**−0.34**	**−0.34**	**0.80**	-					
27. W2 HP	**0.21**	**−0.07**	**−0.20**	**−0.21**	**−0.21**	**−0.17**	**−0.16**	**−0.24**	**−0.26**	**−0.29**	**−0.25**	**−0.24**	**−0.23**	**−0.27**	**−0.25**	**−0.26**	**−0.23**	**−0.21**	**−0.28**	**−0.30**	**−0.32**	**−0.28**	**−0.33**	**−0.33**	**0.74**	**0.72**	-				
28. W2 MAT	**0.28**	**−0.06**	**−0.23**	**−0.25**	**−0.20**	**−0.16**	**−0.17**	**−0.26**	**−0.28**	**−0.31**	**−0.28**	**−0.27**	**−0.27**	**−0.30**	**−0.26**	**−0.29**	**−0.25**	**−0.24**	**−0.31**	**−0.32**	**−0.34**	**−0.27**	**−0.36**	**−0.36**	**0.93**	**0.94**	**0.88**	-			
29. W2 SCS	**0.05**	0.01	**0.06**	**0.05**	**0.09**	**0.11**	**0.05**	**0.09**	**0.13**	**0.17**	**0.16**	**0.14**	**0.16**	**0.17**	**0.18**	**0.13**	**0.21**	**0.17**	**0.21**	**0.21**	**0.14**	**0.16**	**0.12**	**0.20**	**0.13**	**0.15**	**0.09**	**0.14**	-		
30. W2 SOO	**0.13**	**−0.07**	**−0.17**	**−0.16**	**−0.19**	**−0.17**	**−0.16**	**−0.22**	**−0.16**	**−0.17**	**−0.16**	**−0.17**	**−0.15**	**−0.17**	**−0.16**	**−0.19**	**−0.15**	**−0.14**	**−0.14**	**−0.17**	**−0.21**	**−0.20**	**−0.18**	**−0.21**	**0.42**	**0.38**	**0.43**	**0.45**	**0.32**	-	
31. W2 EGO	**0.11**	*−0.03*	**−0.07**	**−0.07**	**−0.06**	*−0.04*	**−0.07**	**−0.08**	−0.02	0.01	0.00	−0.02	0.01	0.00	0.01	*−0.03*	**0.04**	0.02	**0.04**	*0.03*	**−0.04**	−0.02	*−0.03*	0.00	**0.33**	**0.33**	**0.32**	**0.36**	**0.82**	**0.81**	-
Mean	13.1	-	4.2	4.0	4.0	4.4	3.8	4.1	5.1	5.2	4.8	5.0	4.7	5.0	5.1	4.9	5.1	4.9	4.6	4.9	4.9	5.2	5.5	5.0	2.2	2.4	1.7	2.1	3.7	2.2	2.9
SD	1.32	-	0.87	0.96	0.84	0.91	1.1	0.73	0.96	0.95	0.96	1.0	1.1	0.99	0.90	0.92	0.93	1.1	1.0	1.1	1.1	0.87	1.4	0.83	1.2	1.3	0.92	1.0	1.1	1.1	0.90
α	-	-	0.93	0.91	0.69	0.69	0.80	0.95	0.89	0.90	0.88	0.85	0.88	0.91	0.84	0.82	0.86	0.67	0.88	0.84	0.88	0.82	0.93	0.98	0.90	0.86	0.85	0.95	0.75	0.90	0.85
Mean inter-item correlation	-	-	0.53	0.53	0.28	0.45	0.57	0.36	0.58	0.61	0.53	0.58	0.56	0.64	0.51	0.44	0.55	0.51	0.51	0.63	0.61	0.49	0.65	0.42	0.54	0.57	0.54	0.49	0.34	0.51	0.31

Note. Correlation coefficients in **bold** and in *italic* are significant at 0.01 and 0.05 levels, respectively. W1 = Wave 1, MU = mutuality, COM = communication, CONF = conflict, PCOC = parental concern, PCOT = parental control, FF = family functioning, BO = bonding, RES = resilience, SC = social competence, RPB = recognition for positive behavior, EC = emotional competence, CC = cognitive competence, BC = behavioral competence, MC = moral competence, SD = self-determination, SE = self-efficacy, CPI = clear and positive identity, BF = beliefs in the future, PI = prosocial involvement, PN = prosocial norms, SP = spirituality, PYD = positive youth development, AC = acquisition centrality, MP = materialistic possession, HP = hedonistic pursuit, MAT = materialism, SCS = self-conceit, SOO = self over others, EGO = egocentrism, W2 = Wave 2, ^a^ 1 = male, 2 = female.

**Table 2:** Results of mediation analyses with controlling for age and sex (*n* = 4922).

Pathways	Standardized Effects	95% CI (Lower Bound)	95% CI (Upper Bound)	Percentage of Indirect Effect in the Total Effect (%)	R^2^ (%)
**Materialism**					
*Direct effects*					
Family functioning → positive youth development	0.347	0.318	0.376	43.4	19.8
Positive youth development → materialism	−0.281	−0.328	−0.234		
Family functioning → materialism	−0.128	−0.171	−0.085		
*Indirect effect*					
Family functioning → positive youth development → materialism	−0.098	−0.118	−0.078		
*Total effect*					
Family functioning → materialism	−0.226	−0.267	−0.185		
**Egocentrism**					
*Direct effects*					
Family functioning → positive youth development	0.347	0.318	0.376	81.6	15.7
Positive youth development → egocentrism	−0.356	−0.401	−0.311		
Family functioning → egocentrism	−0.028	−0.218	0.162		
*Indirect effect*					
Family functioning → positive youth development → egocentrism	−0.124	−0.177	−0.071		
*Total effect*					
Family functioning → egocentrism	−0.152	−0.348	0.044		

Note: The effect is significant (*p* < 0.05) when 95% CI does not contain zero.

## Data Availability

The data presented in this study are only available on request from the corresponding author.
